# Practical Diagnosis

**Published:** 1903-01

**Authors:** 


Practical Diagnosis. The Use of Symptoms and Physical Signs in the Diagnosis of Disease By Hobart Amory Hare, M. D., B. Sc., Professor of Therapeutics in the Jefferson Medical College of Philadelphia; Physician to the Jefferson Medical College Hospital; Author of a Textbook of Practical Therapeutics. Fifth edition, revised and enlarged. Octavo, pp. 710. Illustrated. Philadelphia and New York: Lea Brothers & Co. 1902. [Price, cloth, $5.co net.]

This book is manifestly the work of an experienced teacher. The method of teaching clinical medicine is the logical interpretation of the history of the patient and connecting it with the signs and symptoms discovered. The eliciting of the signs and the search for symptoms is methodically shown.

In private practice, as in hospital service, there is only one way of diagnosis, and that is by grouping of symptoms and signs until the clinical picture is complete, and that clinical picture we label a disease. The book is divided into two parts. The first treats of the manifestation of disease in organs. The first chapter takes up successively the appearance of the face and extremities, and includes deformities, spasms, tremors and deformities.

The remaining chapters are a close study of the tongue, skin, thorax and its viscera, the abdomen and its viscera, the blood-vessels and the eye.

The chapter on the examination of the blood and the interpretation of the various changes noted, also the chapters on the examination of urine and feces, are very valuable and are clearly written.

The second part is a study of the manifestation of disease by symptoms and presents in succession the significance of fever, headaches, vertigo, coma, convulsions, hiccough, vomiting, pain, tendon reflexes, and the changes in speech.

The text is in the author's well-known clear and forceful style. The deductions from symptoms are logical. The understanding of the various areas of anesthesia of nerve distribution of regional disease is made easy by illustrations admirably adapted for the purpose. The book is one of the most helpful works on diagnosis published. J. W. P.

